# The protein quality control system in motoneuron diseases

**DOI:** 10.1186/2193-1801-4-S1-L55

**Published:** 2015-06-12

**Authors:** Angelo Poletti, Paola Rusmini, Valeria Crippa, Riccardo Cristofani, Maria Elena Cicardi, Marco Meroni, Rita Galbiati, Margherita Piccolella, Elio Messi

**Affiliations:** Università degli Studi di Milano, Italy

**Keywords:** Motoneuron diseases, protein misfolding, autophagy

Spinobulbar muscular atrophy (SBMA) and amyotrophic lateral sclerosis (ALS) are two related motorneuron diseases (MNDs), both characterized by the presence of inclusions o aggregates of proteinaceous materials. In SBMA, aggregates contain mutant androgen receptors (AR) with an elongated polyglutamine tract (ARpolyQ), while in ALS aggregates contain TDP43, ubiquilin, optineurin, etc. Exceptions are familial ALS (fALS) forms linked to superoxide dismutase 1 (SOD1) mutations, in which aggregates are composed of mutant SOD1. Protein aggregation occurs when a large excess of proteins with aberrant conformations (misfolding) in produced and poorly cleared from the cells. Neurons contains an efficient protein quality control (PQC) system, but this may be insufficient to correctly remove misfolded proteins, especially during aging. The PQC system requires the activities of efficient chaperones and of the two major intracellular degradative systems: the ubiquitin-proteasome (UPS) and the autophagic systems. When misfolded protein are recognized by chaperones, they can be removed via autophagy by their engulfment into autophagosomes which then fuseto lysosomes. We found that motoneurons may responds to misfolded species by activating the expression of a small HSP, HSPB8, which facilitate the clearance of misfolded species via autophagy, usually acting by restoring the proper autophagic flux, found altered in MNDs. HSPB8 requires its co-chaperone BAG3. BAG3 binds the protein 14-3-3 and with this it interacts with dynein in a complex which also includes HSC70-CHIP. Dynein moves this large complex on the microtubules organization center where autophagosomes are assembled. Here, CHIP ubiquitinated misfolded protein substrates allowing their recognition by p62 and clearance from the motoneurons. Thus, together, the PQC and the HSPB8 proteins help to protect motoneurons from damages associated to the presence of aberrant protein species accumulating in affected cells.

